# Lactate metabolism coordinates macrophage response and regeneration in zebrafish

**DOI:** 10.7150/thno.65235

**Published:** 2022-05-13

**Authors:** Candice Bohaud, Jholy De La Cruz, Claudia Terraza, Audrey Barthelaix, Béryl Laplace-Builhé, Christian Jorgensen, Yoan Arribat, Farida Djouad

**Affiliations:** 1 IRMB, Univ Montpellier, INSERM, Montpellier, France; 2 CHU Montpellier, Montpellier, F-34295 France

**Keywords:** Regeneration, lactate, macrophage, zebrafish, single cell RNA-sequencing

## Abstract

**Rationale:** Macrophages are multifunctional cells with a pivotal role on tissue development, homeostasis and regeneration. Indeed, in response to tissue injury and the ensuing regeneration process, macrophages are challenged and undergo massive metabolic adaptations and changes. However, the control of this metabolic reprogramming by macrophage microenvironment has never been deciphered in vivo.

**Methods:** In this study, we used zebrafish model and caudal fin resection as a robust regeneration system. We explored specific changes in gene expression after tissue amputation via single-cell RNA sequencing analysis and whole-tissue transcriptomic analysis. Based on the identification of key modifications, we confirmed the role of the lactate pathway in macrophage response and fin regeneration, through the combination of chemical and genetic inhibitors of this pathway.

**Results:** Single cell RNA sequencing revealed the upregulation of different genes associated with glycolysis and lactate metabolism in macrophages, upon fin regeneration. Hence, using chemical inhibitors of the LDH enzyme, we confirmed the role of lactate in macrophage recruitment and polarization, to promote a pro-inflammatory phenotype and enhance fin regeneration. The genetic modulation of monocarboxylate transporters illustrated a complex regulation of lactate levels, based on both intracellular and extracellular supplies. Commonly, the different sources of lactate resulted in macrophage activation with an increased expression level of inflammatory cytokines such as *TNFa* during the first 24 hours of regeneration. Transcriptomic analyses confirmed that lactate induced a global modification of gene expression in macrophages.

**Conclusion:** Altogether, our findings highlight the crucial role of lactate at the onset of macrophage differentiation toward a pro-inflammatory phenotype. The deep modifications of macrophage phenotype mediated by lactate and downstream effectors play a key role to coordinate inflammatory response and tissue regeneration.

## Introduction

Among vertebrates, some species are capable of fully regenerating their tissues such as heart, jaw, limb and/or fin after an extensive insult. The process that allows the functional restoration of complex structures after amputation or tissue injury is called epimorphic regeneration. This mechanism is initiated by the formation of a transient structure called blastema, a heterogeneous mass of cells mainly composed of highly proliferative mesenchymal cells that ultimately differentiate into several distinct cell types and give rise to a perfect copy of the original amputated structure 1, 2. The role of macrophages in epimorphic regeneration has been extensively explored by ourselves and by others. Originally described for their capacity of phagocytosis, macrophages have been revealed to have a pivotal role in inflammation and its resolution. In regenerative species such as zebrafish, macrophages play a crucial role in controlling appendage regeneration after amputation 3, 4. Indeed, genetic or pharmacologic depletion of macrophages at different time points of the regeneration process confirmed that an absence of early recruited macrophages represses this process 3, 4. To complete their functions, macrophages dynamically answer to change in their microenvironment and adapt through different stages of activation. We recently illustrated this plasticity and evidenced the sequential accumulation of distinct macrophage subtypes within the regenerating caudal fin of the zebrafish larvae. *TNFa*-expressing macrophages are particularly important to orchestrate fin regeneration 4.

Answering to microenvironmental changes, macrophages are submitted to a metabolic reprogramming that guarantees their role in homeostasis and inflammation 5. In vitro approaches illustrated that pro-inflammatory macrophages (M1-like) preferentially use a glycolytic metabolism while anti-inflammatory macrophages (M2-like) rather rely on the metabolism of oxidative phosphorylation 6-8. The metabolic phenotype of macrophage subpopulations was also recently characterized *in vivo*, in zebrafish, during an inflammatory response 9. However, the time course of macrophage metabolic reprogramming during regeneration process and its function remain unknown. Although a recent study showed that the process of caudal fin regeneration relies on global metabolic reprogramming, the metabolic changes of blastemal macrophages as well as their role in regeneration have not been investigated. 10.

In the present study, we relied on the caudal fin regeneration mode, in 3 days post-fertilization (3 dpf) zebrafish larvae to perform single-cell RNA sequencing (scRNA-seq) approach and to identify changes in the cells composing the regenerative tissue. Interestingly, we revealed key modifications in macrophage expression profile, in particular in genes associated with glycolysis and lactate metabolism. Hence, combining chemical and genetic repressions of key regulators, we confirmed the impact of the glycolysis and lactate production on (i) macrophage recruitment/accumulation at the wound site, on (ii) macrophage activation/polarization, on (iii) blastema formation and zebrafish fin regeneration. Eventually, we showed that the lactate pathway impacts the global expression profile of macrophage. Collectively, our findings give a new insight into the metabolic regulation of macrophage during regeneration.

## Materials and Methods

### Ethics

Animal experimentation procedures were carried out according to the European Union guidelines for handling of laboratory animals (http://ec.europa.eu/environment/chemicals/lab_animals/home_en.htm) and were approved by the Direction Sanitaire de l'Hérault and Comité d'Ethique pour l'Expérimentation Animale under reference APAFIS#2021072114172657 #32511 v2.

### Zebrafish lines, maintenance and handling

Fish and embryo maintenance, staging and husbandry were performed as previously described 4, 11. Embryos were obtained from the University of Montpellier. Experiments were done using wild type strain and the transgenic line *Tg(mpeg1:mCherry-F)* to visualize macrophages 12, and *Tg(mpeg1:mCherry-F;tnfa:eGFP-F)* 13 to visualize *tnfa* expression in macrophages. Embryos were obtained from adult zebrafish pairs by natural spawning and were raised at 28.5 °C in zebrafish tank water.

### Zebrafish larvae manipulation for regeneration assays

Caudal fin amputation was performed on 72 hours post-fertilization (hpf) larvae, as previously described 13. Larvae were anaesthetized in embryo medium supplemented with 0.016% Tricaine and the caudal fin was amputated with a scalpel at the limit of the notochord posterior end.

### Pharmacological modulation of glycolysis and lactate metabolism in zebrafish larvae

2-Deoxyglucose (2DG) (Sigma-Aldrich D6134, 10), stock solution, was dissolved to 1 M in water. A 50 μM solution was then used to carry out the immersion experiments, diluted directly in the fish water after amputation and maintained with different time windows: from 0 to 24 hpA, from 24 to 72 hpA or throughout the duration of the experiments. This solution has also been injected into the caudal vein of larvae at 3 days post-fertilization (dpf), 1 hour before amputation. Larvae untreated or injected with PBS were used as negative controls. Sodium dichloroacetate (DCA) (Santa Cruz 10SC 203275A, 14), stock solution, was prepared at a concentration of 1 M in water. A solution of 500 μM was then used to carry out the experiments in immersion, diluted directly in the fish water after amputation and maintained with different time windows: from 0 to 24 hpA, from 24 to 72 hpA or throughout the duration of the experiments. This solution was also injected, when indicated, into the caudal vein of larvae at 3 dpf, 1 hour before amputation. Larvae untreated or injected with PBS were used as negative controls. Galloflavin (GAL) (Tocris Bioscience, 15), stock solution was prepared at 5 mM in DMSO. A 500 nM solution was then used to carry out the immersion experiments, diluted directly in the fish water after amputation and maintained at different time windows: from 0 to 24 hpA, from 24 to 72 hpA or throughout all regeneration process. This solution was also injected, when indicated, into the caudal vein of larvae at 3 dpf, 1 hour before amputation. Larvae untreated or injected with PBS were used as negative controls. Sodium oxamate (OXA) (02751 Sigma, 16), stock solution was prepared at 1M in DMSO. A 10 mM solution was then used to carry out the immersion experiments, diluted directly in the fish water after amputation and maintained throughout all regeneration process. This solution was also injected, when indicated, into the caudal vein of 3 dpf larvae 1 hour before amputation. Untreated larvae were used as negative controls. Lactate (Sodium L-lactate L7022) (Sigma), stock solution was prepared at 1 M in water. A 100 μM solution was then used to carry out the immersion experiments, diluted directly in the fish water after amputation and maintained throughout the duration of the experiments. Larvae untreated were used as negative controls.

### Genetic modulation of glycolysis and lactate metabolism in zebrafish larvae

The main isoforms of MCT1 (NM_200085) and MCT4 (NM_212708) described by Tseng and colleagues 19 were targeted by CRISPR/Cas mutation, following the procedure described by Kroll and colleagues 20. Briefly, three guideRNAs targeting distinct exons were designed for each gene on https://chopchop.cbu.uib.no/: gRNA1: GGTGTTCTTCAAGGAGATTG, gRNA2: CGGCCAATCATGATCGCTGG, gRNA3: TTTGCCACCAGACCCATAGA for MCT1 and gRNA1: CTTATCCGGGAGTTTGGAGT, gRNA2: GACACCCAAATCGGTTCACT, gRNA3: TTCACCGTCTTCAAAGATCG for MCT4. Scrambled sequences were used as control 20. For each targeted gene, the mix of the three gRNAs (20 pMol/µl each) with True Cut Cas9 protein v2 (invitrogen, A36496, 500 ng/µl) was injected in zebrafish one-cell zygote (1nl per egg) with Eppendorf microinjector. No letality was reported in crispant embryos. A summary of gRNA sequence and mapping was reported on Figure S5.

### Imaging of zebrafish larvae and quantification

For imaging, larvae were anesthetized in 0.016% Tricaine, immobilized in 35 mm glass bottom dishes (FluoroDish^TM^, World Precision Instruments) using 1% low melting point agarose (Sigma-Aldrich) and covered with a small volume of fish water containing tricaine. Epifluorescence microscopy was used to measure the length and the area of regrowth and performed with a Leica DMI6000CS equipped with a Leica K5 camera. Regenerative fin growth length was evaluated by measuring the distance between the amputation plane and the edge of the fin in the median plane with Fiji. Regenerative fin fold area was measured as the fold area between the notochord and the edge of the fin using the polygon tool of Fiji.

Confocal microscopy was employed to study macrophage behavior, recruitment and activation, using an inverted confocal microscope TCS SP5 and TCS SP8 MP on the Cartigen Platform from IRMB (Leica Microsystems). Images were taken in a sequential mode by frame and processed using Fiji, compressed into maximum intensity projections and cropped. Brightness, contrast, and color levels were adjusted for maximal visibility. The migratory behavior of macrophages was observed at the fin tip with *Tg(mpeg1:mCherry-F)* at 6 hpA, and the recruitment and activation of macrophages were tracked with *Tg(mpeg1:mCherry-F/tnfa:eGFP-F)* at 6 and 24 hpA. Recruited and activated macrophages were counted directly on microscopy images using Fiji.

### Zebrafish larva cell proliferation

Proliferative cells were labelled using immunodetection with anti-phosphorylated histone 3 antibody (H3P). At 6 and 24 hpA, larvae were fixed in 4% paraformaldehyde overnight at 4 °C and stained as previously described 13 using the Rabbit anti-H3P antibody (Cell Signaling). Positive cells in the fin region were quantified on confocal images using Fiji software.

### Single-Cell RNA-seq Data Analysis

For data integration, raw count matrices and metadata generated by our laboratory were imported from the Gene Expression Omnibus repository file GSE158851 21. Feature-barcode matrices were generated for each condition in Rstudio. Downstream analysis- including quality filtering, data normalization and scaling, highly variable genes identification, dimensionality reduction, clustering algorithm and the differential expression testing- was performed using Seurat 4.0.3 packages 22, 23.

For the quality control and filtering, first to avoid low-quality cells, we excluded cells with feature counts less than 200 or over 4000. We then filtered out doublets by using DoubletFinder tool 24. Finally, we excluded the cells expressing more than 10% of transcripts expressed from mitochondrial genes. After quality control, the total numbers of cells were of 878 for the uncut condition and 762 for the cut condition with 15411 detected genes in both conditions (Figure S1D).

For the normalization, Scaling and Dimensional Reduction, we analyzed the integrated samples and associated labels for each condition. This allowed us to keep the same parameters throughout the whole analysis. Data normalization and scaling was performed on the integrated object with the default methods. Find Variables Features function was used to find 2000 highly variable features from cell to cell. Linear dimensional reduction was then carried out through the Principal Component Analysis (PCA) method. Using the ElbowPlot function we determined 16 PCs as the number of principal components explaining the best our data. To visualize data we used the Uniform Manifold Approximation and Projection method (UMAP) 25. For the clustering, we used the Louvain algorithm with a resolution of 1 26. Then, we identified the cell types from the different clusters based on the top marker expression found by the FindAllmarkers function as well as FindMarkers function-based comparisons between close clusters. We also explored shared expression of markers of different cell types from the literature and ZFIN database 27. Myeloid cluster subclustering analysis was performed based on the expression of macrophages (*mpeg1* and *mfap4*) and neutrophils (*lyz* and *mpx*) markers, using the subset function to split macrophages and neutrophils. For differential expression analysis, an additional filtering was performed on the macrophages considering only macrophages with a positive expression of mpeg1.1 since this is the marker used for further macrophages quantification and behavior characterization. Differential expression, was then performed by using the Seurat Findmarkers function with min.pct = 0 and logfc.threshold = 0.25 as parameters as well as specifying the DESeq2 method 28. We considered a significant differential expression based on q-values < 0.05 for the cluster markers and p-values < 0.01 for the cut vs uncut comparisons. For the ingenuity pathway analysis, our list of metabolic genes containing their foldchanges and p-values from the differential expression testing was uploaded to the IPA system (version 65367011, Ingenuity Systems; Qiagen). We then performed gene expression-based core analysis which included canonical pathway analysis and molecular network generation. IPA uses an algorithm which generates networks from the up-loaded data and databases information. The scores of each network were generated based on hypergeometric distribution, where the significance is obtained by Fisher's exact test. For canonical pathway analysis, the -log (P-value) > 2 was taken as threshold, the Z-score > 2 was defined as the threshold of significant activation, whilst Z-score < -2 was defined as the threshold of significant inhibition 28.

### RNA extraction, array hybridization and data processing

Tissues for microarray data generation consisted of three biological replicates from each of 2 conditions: control and the lactate-treated samples (24 hpa macrophages from 150 caudal fins for each replicate). Total RNA was isolated from all samples using RNeasy Mini Kit (Qiagen). The amount and purity of extracted RNA were determined using a NanoDrop ND-1000 spectrophotometer (NanoDrop ND, Termo Fisher Scientifc) as well as their integrity by using the Agilent 2100 Bioanalyzer (Agilent Technologies; http://agilent.com/). cDNA generation, amplification, fragmentation and biotinylation were performed using the Ambion WT Expression Kit (Ambion, Austin, TX, USA). Microarray experiments took place at the IRMB (Montpellier University Hospital) DNA microarray platform. All samples were hybridized to Affymetrix Zebrafish 1.0ST Arrays according to Affymetrix recommendations. Data was acquired on a GeneChip® Scanner 3000 and CEL file generation was performed using AGCC.

### Microarray analysis

CEL files were processed and analyzed in Rstudio following the Klaus and Reisenauer analytical workflow 29. Probe intensity data was extracted, normalized and summarized at the core level with Robust Multi-chip Average (RMA) method from oligo package.

Differential expression analysis was performed between control and lactate-treated samples by building a linear model using limma package. Genes were considered as differentially expressed if a P-value < 0.05 was displayed. Among the DEGs, top 25 and bottom 25 genes based on the foldchange were selected to plot the heatmap for lactate signature effect. Scaled expression values were used to plot the heatmaps (z-score). Gene Ontology enrichment analysis was performed using DAVID platform 30.

### Statistical analyses

Graph Pad Prism 6.0 Software (San Diego, CA, USA) was used to generate the graphs and to analyze the data presented in all the figures. Specific statistical tests were used to evaluate the significance of differences between the groups. Graphs show the mean ± standard error of the mean (SEM).

## Results

### Identification of key metabolic pathways activated in macrophages during regeneration

**Figure 1:**
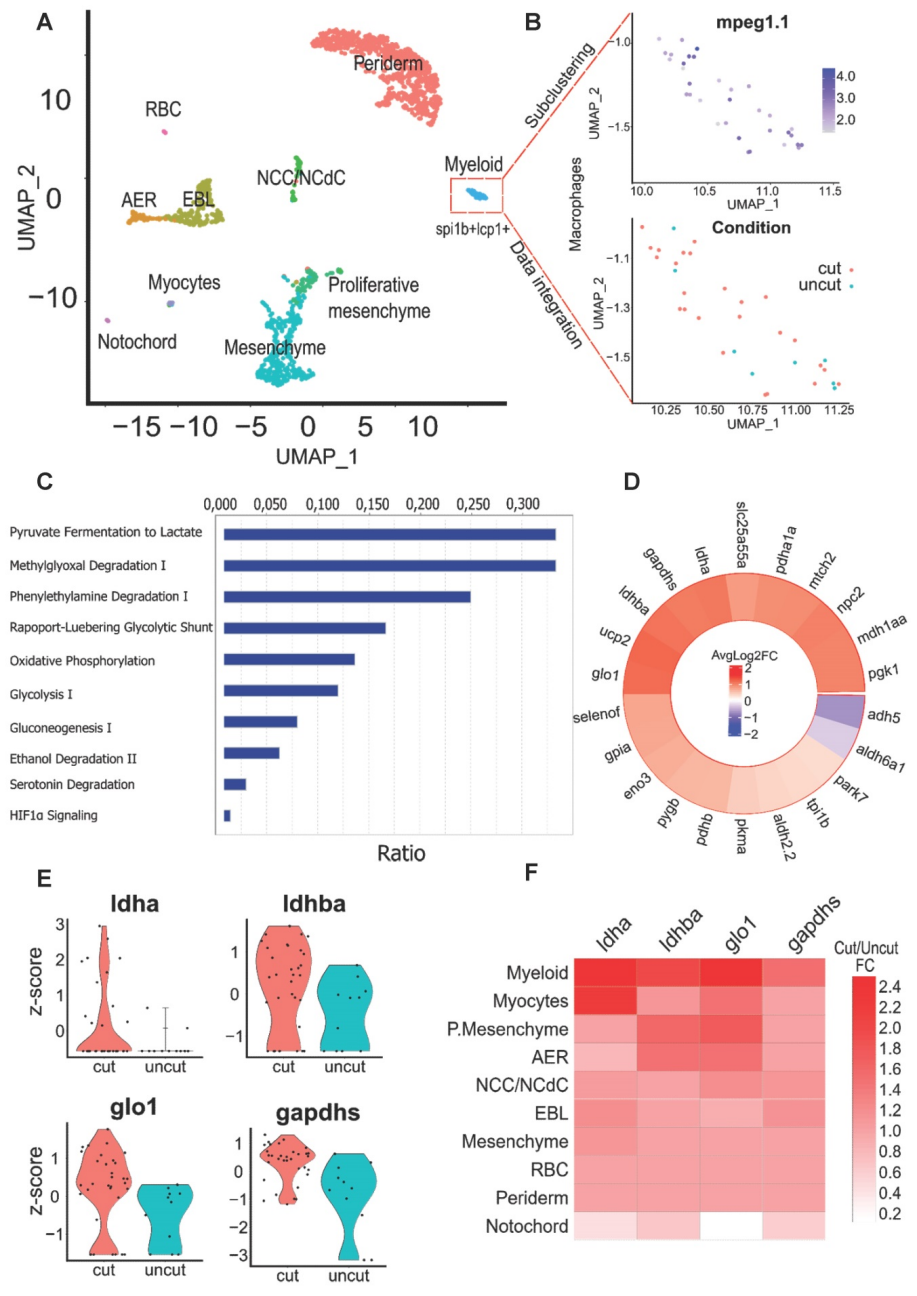
** Identification of key pathways modulated within macrophages of the regenerating caudal fin from Single-cell RNAseq data.** (A) Clustering of the integrated data from the 24 hpA caudal fin (cut) and their uninjured counterparts (uncut). AER: apical ectodermal ridge, EBL: epithelial basal layers, NCC/NCdC: Neural crest cells/NCC derived cells. (B) Feature plot of macrophages subset based on *mpeg1.1* expression and dimensional reduction plot of their distribution through the cut and uncut conditions. (C) Canonical pathway analysis from the Ingenuity Pathway analysis (IPA) of the metabolic network. Ratio (genes detected / total number of genes of the pathway) within macrophages subset. (D) Heatmap of the cut/uncut gene expression ratio of the main glycolysis associated-genes (E) Violin plots of the top differentially expressed glycolytic and lactate-associated genes within cut and uncut conditions. (F) Heatmap of the cut/uncut expression fold change for the top DE genes through the different caudal fin cell populations.

Amputation of the caudal fin fold in 3dpf larvae induces a robust regeneration process leading to the restoration of the original tissue within 3 days. This process is referred as epimorphic regeneration and depends on the formation of a blastema at 24 hours post-amputation (hpA). In order to identify modifications of transcriptomic profiles in a cell-specific manner, we compared intact (uninjured) and regenerating caudal fin at 24 hpA by scRNA-seq and Chromium system (10X Genomics platform) 21. Cell clustering of an integrated dataset of regenerating and uninjured fin, based on the Louvain unsupervised clustering algorithm, identified 10 clusters of cells (Figure 1A). Among them, myeloid and mesenchymal clusters exhibited important modifications on the number of cells present after amputation. The recruitment of injury-induced macrophages and the removal of mesenchymal tissue after fin amputation, which is not restored until later phases, might explain these observations (Figure S1A). Characteristic markers of myeloid cells including *Spi-1 proto-oncogene b* (*spi1b*), *coronin1a* (*coro1a*) and *l-plastin* (*lcp1*), were strongly expressed in the myeloid cluster. Within this cluster, sub-clustering was performed by using *myeloid-specific peroxidase* (*mpx) and lysozyme* (*lyz*) markers expression in order to exclude the few neutrophils that had not yet left the fin by reverse migration 32 and focus our attention on macrophages expressing *mpeg1* and *mfap4* exclusively (Figure 1B, Figure S1B). Then, we performed differential gene expression analysis focusing on the macrophage population. To that end, we compared the expression data related to cell metabolism between uninjured and injured samples (Figure 1C-D). Canonical pathway analysis with Ingenuity Pathway Analysis (IPA) based on the list of differentially expressed genes identified an enrichment in genes associated with the lactate pathway, specifically in macrophages. Indeed, 33% of the molecular components triggering lactate production were differentially expressed in amputated tissue compared with intact fin, suggesting a key role of lactic fermentation pathway in macrophages during regeneration (Figure 1C). Consistently, methylglyoxal degradation, described to promote lactate production as a strategy to manage strong glycolytic activity 33, was also among the most enriched pathways in macrophages during regeneration.

We focused on the differential expression upon amputation of glycolytic genes, carefully selected from the literature and databases, within the macrophage cluster. We identified 4 targets: *glyoxalase 1* (*glo1*), the main enzyme in the glyoxalase system, g*lyceraldehyde-3-phosphate dehydrogenase* (*gapdh*), a key glycolytic enzyme, *lactate dehydrogenase B* (*ldhb*) subunit of the LDH complex, involved in the pyruvate interconversion to lactate among the top 5 differentially expressed genes based on p-value. Moreover, *lactate dehydrogenase A* (*ldha*) another LDH subunit displaying a strong lactate production activity as well as the previous enzymes were found within the top 15 genes with the highest fold change expression in regenerative conditions.

Of note**,** *alcohol dehydrogenase 5* (*adh5*) and *aldehyde dehydrogenase 6* (*aldh6a1*) were among the few down-regulated genes of the list suggesting an inhibition of alcoholic fermentation from pyruvate (Figure 1D).

Violin plot visualization confirmed the differential gene expression levels of these enzymes during caudal fin regeneration (Figure 1E). While exploring lactate genes throughout the different cell clusters, we found that lactate associated enzymes displayed an enrichment within the myeloid cluster and in particular *ldha* subunit which was almost exclusively expressed in the myeloid cluster (Figure 1F). We also compared the expression profile of these enzymes in the neutrophils still present in the blastema 24 hpA and did not see any difference of expression between amputated and intact tissue, highlighting a macrophage-specific mechanism (data not shown). Using IPA, we generated a network of up- and down-regulated genes specifically in the regenerating fin as compared to the intact one. We identified a set of glycolytic and oxidative phosphorylation (OXPHOS) metabolic actors pointing towards the lactate pathway. Of note, the most interconnected genes in the network were *ldha*, *ldhb*, *pyruvate kinase M (pkm)* and several cytochromes (Figure S1C). These results underline the complexity of the *in vivo* metabolic processes involved during caudal fin regeneration and indicate that although lactate and glycolysis are of importance, OXPHOS, the main metabolic pathway during development process is still actively used. Nevertheless, OXPHOS was widely active among different cell clusters in injured and uninjured conditions. These findings prompted us to decipher the impact of glycolysis and lactate metabolism on regeneration.

### Glycolysis and lactate metabolism are required for macrophage recruitment and polarization during caudal fin regeneration

**Figure 2:**
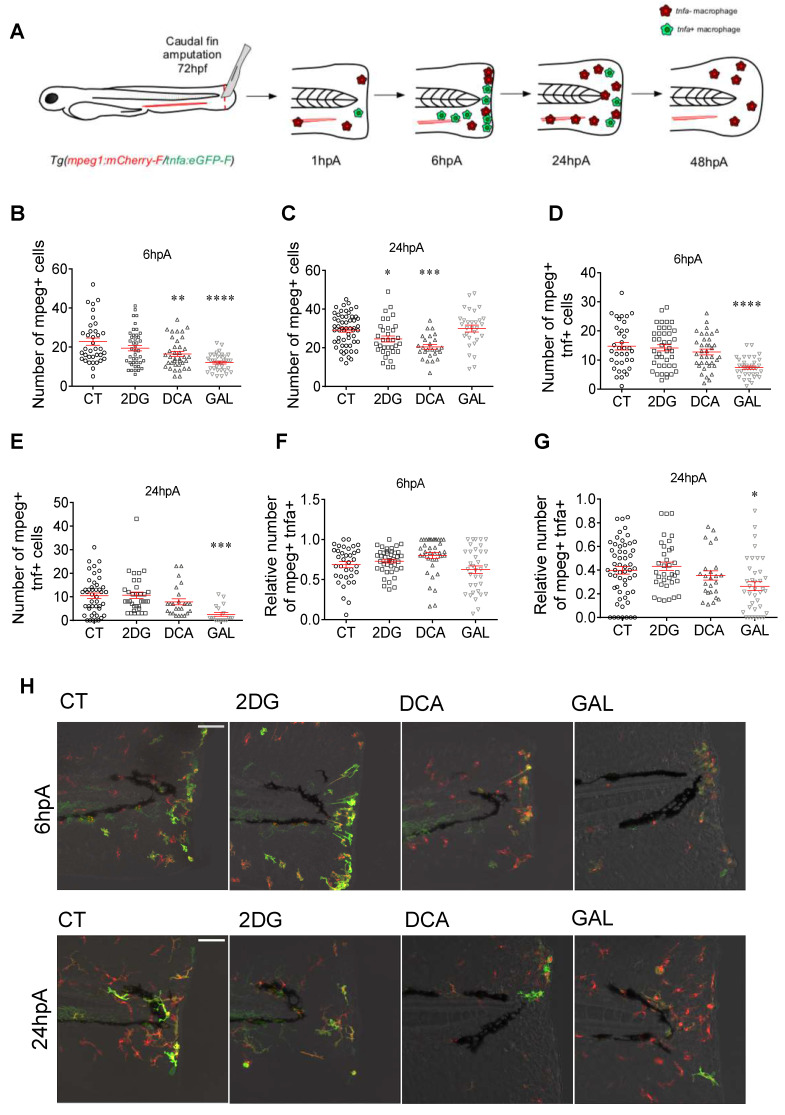
** Glycolytic and lactate inhibitors alter the recruitment and the polarization of macrophages.** (A) Experimental design of macrophage recruitment, migration, and polarization after amputation. (B) Graph showing the quantification of the number of *mpeg*^+^ macrophages in the entire fin at 6 hpA after amputation and immersion with zebrafish water (CT), 2DG (50 μM), DCA (500 μM), or GAL (500 nM) (mean ± SEM, n > 30*,* ordinary one-way ANOVA, Dunnett's multiple comparisons test, compared to control, **p ≤ 0.01, ****p ≤ 0.0001). (C) Graph showing the quantification of the number of *mpeg*^+^ macrophages in the entire fin at 24 hpA after amputation and immersion with zebrafish water (CT), 2DG (50 μM), DCA (500 μM), or GAL (500 nM) (mean ± SEM, *n< 30,* Kruskal-Wallis, Dunn's multiple comparisons test, compared to control, *p ≤ 0.05, ***p ≤ 0.001). (D) Graph showing the quantification of the number of *mpeg*^+^*tnfa*^+^ macrophages in the entire fin at 6 hpA after amputation and immersion with zebrafish water (CT), 2DG (50 μM), DCA (500 μM), or GAL (500 nM) (mean ± SEM, n > 30*,* ordinary one-way ANOVA, Dunnett's multiple comparisons test, compared to control, ****p ≤ 0.0001). (E) Graph showing the quantification of the number of *mpeg*^+^*tnfa*^+^ macrophages in the entire fin at 24 hpA after amputation and immersion with zebrafish water (CT), 2DG (50 μM), DCA (500 μM), or GAL (500 nM) (mean ± SEM, n > 30*,* ordinary one-way ANOVA, Dunnett's multiple comparisons test, compared to control, ***p ≤ 0.001). (F) Graph showing the relative number of *mpeg*^+^*tnfa*^+^ macrophages (fold change of *mpeg*^+^*tnfa*^+^ macrophages over the total number of *mpeg*^+^ macrophages macrophages) in the entire fin at 6 hpA after amputation and immersion with zebrafish water (CT), 2DG (50 μM), DCA (500 μM), or GAL (500 nM) (mean ± SEM, n > 30*,* ordinary one-way ANOVA, Dunnett's multiple comparisons test, compared to control, non-significant). (G) Graph showing the relative number of *mpeg*^+^*tnfa*^+^ macrophages (fold change of mpeg^+^ tnfa*^+^* macrophages over the total number of *mpeg^+^* macrophages) in the entire fin at 24 hpA after amputation and immersion with zebrafish water (CT), 2DG (50 μM), DCA (500 μM), or GAL (500 nM) (mean ± SEM, n > 30*,* ordinary one-way ANOVA, Dunnett's multiple comparisons test, compared to control, *p ≤ 0.05). (H) Z projection of confocal images illustrating macrophage recruitment and activation at 6 and 24 hpA after amputation and immersion with zebrafish water (CT), 2DG (50 μM), DCA (500 μM), or GAL (500 nM) (Scale bar = 60 μm).

In zebrafish 72 hpf larvae, macrophage subpopulations can be monitored in 4-dimension during the regeneration process through the use of transgenic line *Tg(mpeg1:mCherry-F/tnfa:eGFP-F)* 13. While the whole population of macrophages expresses a farnesylated mCherry (mCherry-F), activated cells can be discriminated through the expression of *TNFa*-GFP, pro-inflammatory marker. After caudal fin amputation, macrophages are recruited to the wound site within few minutes, draw a line at 6 hpA, and leave the amputation site around 18 hpA to reach the bloodstream or patrol in the regenerating tissue until 72 hpA (Figure 2A) as previously described 4, 13. To study the role of glycolysis and lactate metabolism on macrophage recruitment and behavior in the regenerating tissue, we focused on macrophages accumulation at the fin tip at 6 hpA, by measuring the total number of *mpeg+* cells at the wound site (Figure 2B-C). We administrated different metabolic inhibitory drugs directly in the fish water: the 2-deoxyglucose (2DG), a glucose analogue that inhibits the first step of glycolysis 34, dichloroacetate (DCA), a pyruvate dehydrogenase kinase inhibitor which increases the entry of pyruvate into the Krebs cycle and indirectly blocks lactate production 35 and galloflavin (GAL), a direct inhibitor of the lactate, blocking the pyruvate catabolic enzyme (LDH) A and B isoforms 36 (Figure S2A). Confocal microscopy analysis at 6 hpA revealed that the macrophage barrier formation in the presence of DCA and GAL were significantly impaired compared to the control and 2DG conditions (Figure S2B-D). This effect was correlated with a decreased number of total macrophages, in the entire fin, at the same time i.e., 6 hpA (Figure 2B and Figure S2B). At 24 hpA, the total number of macrophages in the entire fin was also significantly reduced upon the larva treatment with 2DG and DCA but not with GAL (Figure 2C and Figure S2C).

Pro-inflammatory macrophages expressing *tnfa* accumulated during the first 24 hpA in the regenerating fin and underwent a phenotypic conversion towards a non-inflammatory phenotype or leave the site of injury from 24 hpA to 72 hpA (Figure 2A) 4. To address the role of glycolysis and lactate metabolism on macrophage activation, we grew *Tg (mpeg1:mCherry-F/tnfa:eGFP-F)* larvae in presence of metabolic drugs. Live imaging by confocal microscopy revealed that only GAL significantly decreased the number of macrophages expressing *tnfa* at 6 hpA and 24 hpA (Figure 2D-E, 2H). These results were supported by the quantification of the relative number of macrophages expressing *tnfa* (ratio of *mpeg1*^+^*tnfa*^+^: *mpeg1*^+^ macrophages) showing a decrease at 24 hpA but not at 6 hpA in the presence of GAL (Figure 2F-H).

Altogether, these results show that the inhibition of both glycolysis and lactate metabolism impair the recruitment and the migratory behavior of macrophages within the regenerating fin of zebrafish larvae while only the direct inhibition of the lactate metabolism represses macrophage polarization towards a pro-inflammatory phenotype.

### Inhibition of glycolysis and lactate metabolism impairs regeneration efficiency

**Figure 3:**
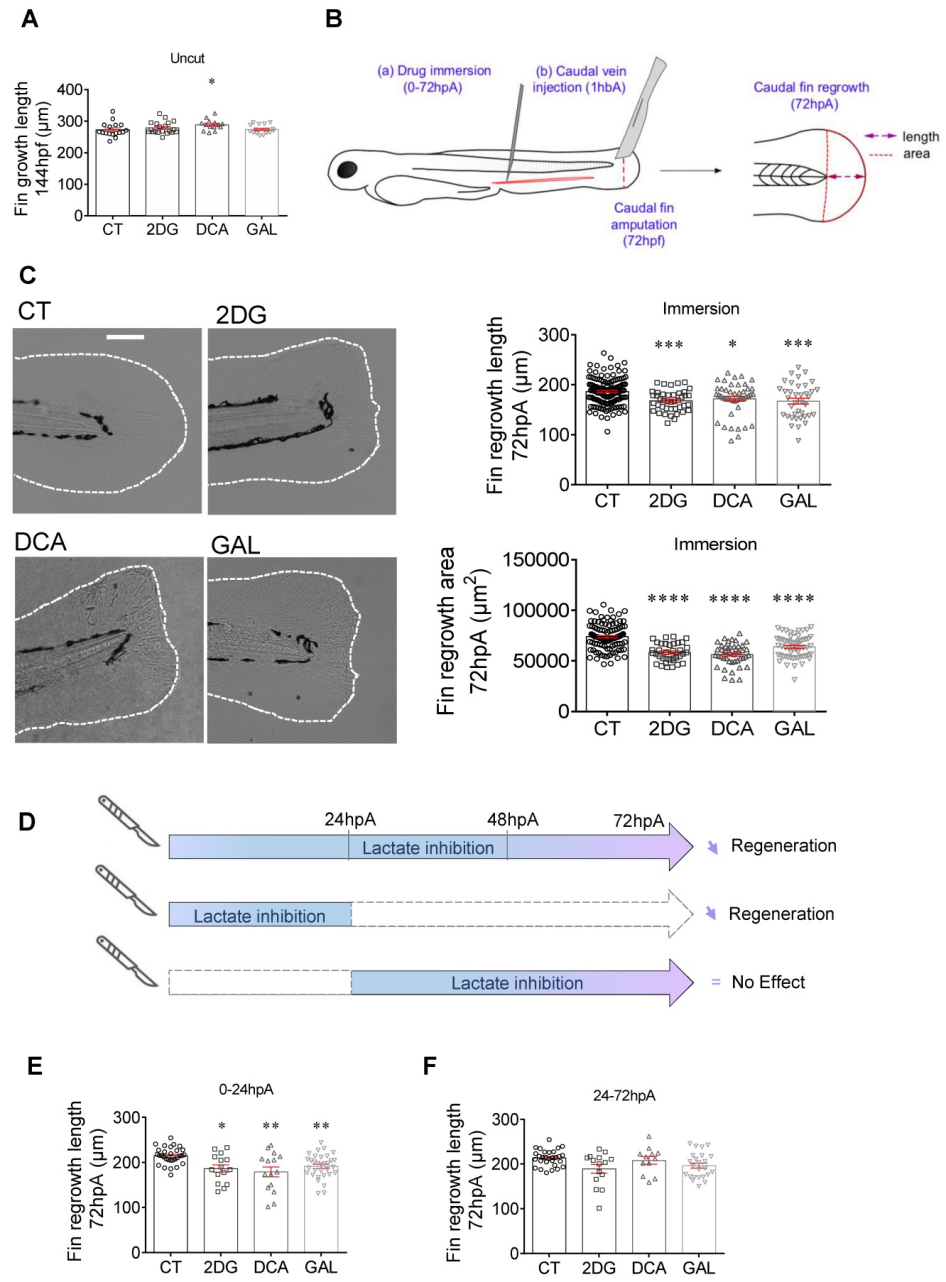
** Glycolytic and lactate inhibitors impact on the caudal fin regeneration after amputation.** (A) Graph showing the fin growth length of uncut larvae at 144 hpf when immersed in zebrafish water (CT), or when immersed in 2DG (50 μM), DCA (500 μM), or in GAL (500 nM) (mean ± SEM, *n < 30,* Kruskal-Wallis, Dunn's multiple comparisons test, compared to control, *p ≤ 0.05). (B) Design of the experiment: (a) add drugs in immersion from 0 until 72 hpA, or (b) add drug in caudal vein injection at 1 hour before amputation (1 hbp), amputation of the caudal fin and measurement of regrowth. (C) Representative images of caudal fin regeneration at 72 hpA (Scale bar = 100 μm) with the corresponding graph showing the fin length or area at 72 hpA, after amputation and immersion with zebrafish water (CT), 2DG (50 μM), DCA (500 μM), or GAL (500 nM) from 0 to 72 hpA (mean ± SEM, n > 30*,* ordinary one-way ANOVA, Dunnett's multiple comparisons test, compared to control, *p ≤ 0.05, ***p ≤ 0.001, ****p ≤ 0.0001). (D) Experimental design illustrating the time windows of drug administration and the impact on fin regeneration. (E) Graph showing the fin length at 72 hpA, after amputation and immersion with zebrafish water (CT), 2DG (50 μM), DCA (500 μM), or GAL (500 nM) from 0 to 24 hpA (mean ± SEM, *n < 30,* Kruskal-Wallis, Dunn's multiple comparisons test, compared to control, *p ≤ 0.05, **p ≤ 0.01). (F) Graph showing the fin length at 72 hpA, after amputation and immersion with zebrafish water (CT), 2DG (50 μM), DCA (500 μM), or GAL (500 nM) from 24 to 72 hpA (mean ± SEM, *n < 30,* Kruskal-Wallis, Dunn's multiple comparisons test, compared to control, non-significant).

We and others evidenced the pivotal role of macrophages in the blastema formation and the regeneration process, in part, through the secretion of pro-inflammatory mediators including TNF-α and IL-1β 4, 39. Indeed, combining macrophage depletion, morpholino knockdown and parabiosis experiments, we have shown that the TNFα/TNFR1 signaling pathway was necessary for blastemal cell proliferation and fin regeneration. To assess the role of glycolysis and lactate metabolism on regeneration, we used the caudal fin transection model of the zebrafish larvae and monitored the blastema formation and the fin regrowth. Fin amputation in 3 dpf larvae led to the formation of a wound epithelium at 6 hpA, a crucial step for the establishment of the blastema, a hyperproliferative structure which appears at 24 hpA to give rise to a novel fin identical to the original one and composed of elongated and well-organized mesenchymal cells at 72 hpA (Figure S3A). Therefore, we first studied the role of 2DG, DCA and GAL on the caudal fin development and did not find any difference in the developmental process of control caudal fin and fin of fish treated with 2DG and GAL at 6 dpf. Of note, a longer caudal fin was observed in the larvae treated with DCA as compared to the control individuals (Figure 3A). This might be due to the fact that the DCA forces the OXPHOS to its maximum and that even though the early stages of organism development with rapid cell proliferation use aerobic glycolysis 40, a gradual increase of oxidative phosphorylation during zebrafish larval development has been described 41.

Then, we evaluated the effect of glycolysis and the lactate on the caudal fin regrowth of amputated 3 dpf larvae maintained in regular water or in drugs containing water. We measured the regrowth length as well as the area of ​​the regenerated fin between the notochord and the fin tip at 72 hpA (Figure 3B) an observed a significant decrease in the fin length and area of the zebrafish exposed to 2DG, DCA and GAL at 72 hpA (Figure 3C). In order to target more specifically the circulating immune cells in particular macrophages, we also injected these drugs into the zebrafish caudal vein and observed a similar inhibitory effect of the tested drugs on caudal fin regeneration as revealed by the significantly reduced fin regrowth in terms of length and area (Figure S3B). To confirm the effect of GAL on the macrophage response (Figure 2) and fin regrowth (Figure 3), we assessed the effect of sodium oxamate (OXA), another LDH inhibitor. We found that OXA also significantly inhibited fin regrowth both when added to the zebrafish water and when injected into the caudal fin (Figure S3C). Then, the effect of 2DG, DCA, GAL and OXA was assessed on the proliferation of blastema cells by H3P immunofluorescence at 24 hpA and showed a significant decrease in blastema cell proliferation in response as compared to the control condition (Figure S3D).

Altogether, these results demonstrate that the general (zebrafish water; immersion) or systemic (zebrafish caudal vein; injection) inhibition of glycolysis and lactate metabolism all along the fin regrowth (i.e., from 0 to 72 hpA) significantly impairs the regeneration potential zebrafish larvae.

Finally, we assessed the effect of lactate inhibition at different time points of the regeneration process. To that end, larvae were incubated in zebrafish water supplemented with or without 2DG, DCA or GAL either during the first 24 hours of the regeneration process corresponding to the pro-inflammatory and proliferation phase of the blastema, or during the late phase of the process i.e., from 24 to 72 hpA, corresponding to the non-inflammatory and cell differentiation phase of the blastema (Figure 3D). We found the addition of 2DG, DCA and GAL in the early phase of the regeneration process significantly impaired the regenerative outgrowth of caudal fins at 72 hpA (Figure 3E), while no significant effect on fin regrowth was observed when the drugs were added in the late phase (Figure 3F). These results reveal the pivotal role of the lactate pathway on caudal fin regeneration in particular during the early phase of the process when blastema forms.

### Extracellular lactate accelerates caudal fin regrowth of the zebrafish larvae

To confirm the impact of lactate through genetic approach, we depleted the main monocarboxylate transporters MCT1 and MCT4 in zebrafish macrophages (Figure S4A-B). While MCT1 represents an important component of lactate uptake, MCT4 ensures its secretion. The depletion of MCT4 by CRISPR strategy did not affect the macrophage activation induced by caudal fin amputation at 24 hpA (Figure S4C-D). Conversely, the lack of MCT1 disrupted the presence of *TNFa*-expressing macrophage at the amputation site (Figure S4C). These results confirmed the importance of lactate for macrophage activation and highlighted the necessity to uptake this substrate from extracellular compartments. The absence of effect in MCT4 crispant suggests that the lactate-secreting cells rely on another transporter to export lactate and activate macrophage upon regeneration. These results call for further investigations to identify macrophage cellular partners and lactate secretory routes.

**Figure 4:**
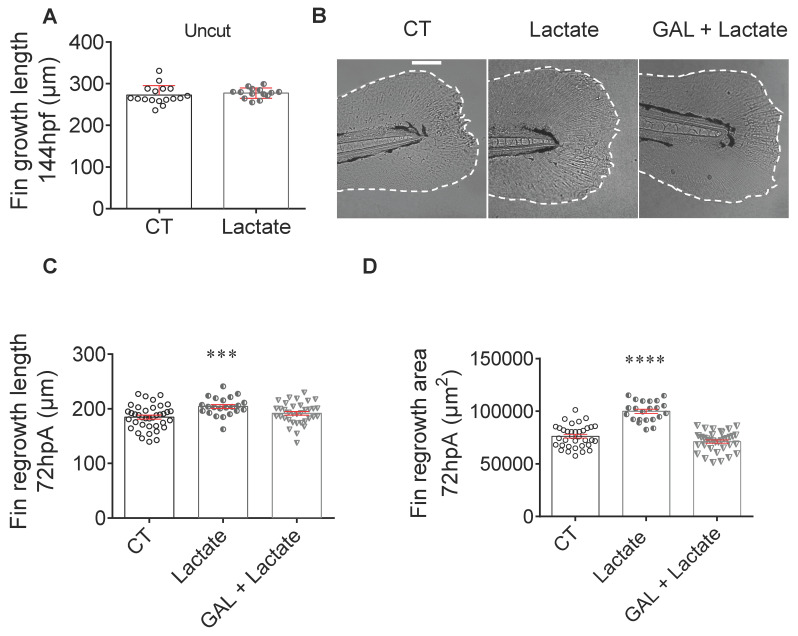
** Exogenous lactate accelerates the caudal fin regeneration after amputation.** (A) Graph showing the fin growth length of uncut larvae at 144 hpf when immersed in zebrafish water (CT), or when immersed in lactate (mean ± SEM, n < 30, Mann Whitney test, two-tailed, non-significant). (B) Representative images of caudal fin regeneration at 72 hpA (Scale bar = 100μm) with the corresponding graph (C) showing the fin length after amputation and immersion with zebrafish water (CT), lactate (100 μM) or GAL (500 nM) and lactate (100 μM) added at the same time (mean ± SEM, n < 30, Mann Whitney test, two-tailed, ***p ≤ 0.001). (D) Graph showing the fin area after amputation and immersion with zebrafish water (CT), lactate (100 μM) or GAL (500 nM) and lactate (100 μM) added at the same time at 72 hpA (mean ± SEM, n < 30, Mann Whitney test, two-tailed, ****p ≤ 0.0001).

To challenge the involvement of this extracellular lactate on regenerative cascade, we supplied amputated larvae with exogenous source of lactate, directly added in fish water. We investigated the potential of fin regeneration in larvae incubated in the presence of exogenous lactate. First, we demonstrated that the lactate exposure had no impact on the normal development of larva caudal fin at 6 dpf (Figure 4A). Then, we assessed whether the lactate complementation can counterbalance the inhibition of endogenous production. Next, we evaluated whether lactate supplementation can counteract the inhibition of endogenous production. We found, that while the inhibition of LDH by GAL reduced fin regrowth (Figure 3C), a concomitant addition of exogenous lactate restored the regenerative properties (Figure 4B-D). Interestingly, exogenous lactate supply went beyond the basal level of regeneration and enhanced fin regrowth compared with control group (Figure 4C-D). This result confirmed the importance of exogenous sources of lactate to promote regeneration. Interestingly, we confirmed a significant increase of H3P positive cells at 6 hpA in the blastema after lactate administration, confirming the increase in blastema cell proliferation under these conditions (Figure S5E).

To confirm a direct effect of exogenous lactate on inflammation, we assessed macrophage activation in presence of 100 µM lactate alone or with 500 μM GAL and determined the impact of lactate on the phenotype observed in the presence of GAL (Figure 2B-2G). Confocal microscopy analysis showed a number of *tnfa* expressing macrophages significantly increased at 6 hpA in regenerating larvae treated with lactate alone or lactate and GAL as compared to untreated larvae (Figure S5A). These results suggested that the early activation of macrophage is mainly driven by an external source of lactate. No effect of the two treatments was observed at 24 hpA (Figure S5B). Quantification of the relative number of *tnfa* expressing macrophages (ratio between *mpeg1^+^tnfa^+^* macrophages and total *mpeg1^+^* macrophages) at 6 hpA showed an increasing trend in larvae treated with lactate alone or lactate and GAL compared to untreated larvae while no effect was observed at 24 hpA (Figure S5C-D).

Altogether, these results confirmed that an external source of lactate can transiently increase macrophage inflammation, accelerate the regeneration process and overcome regrowth impairments due to metabolism inhibition. The combined effects of endogenous production and extracellular import of lactate converge on macrophage activation upon regeneration.

### Lactate induces a global modification of macrophage inflammatory profile

**Figure 5:**
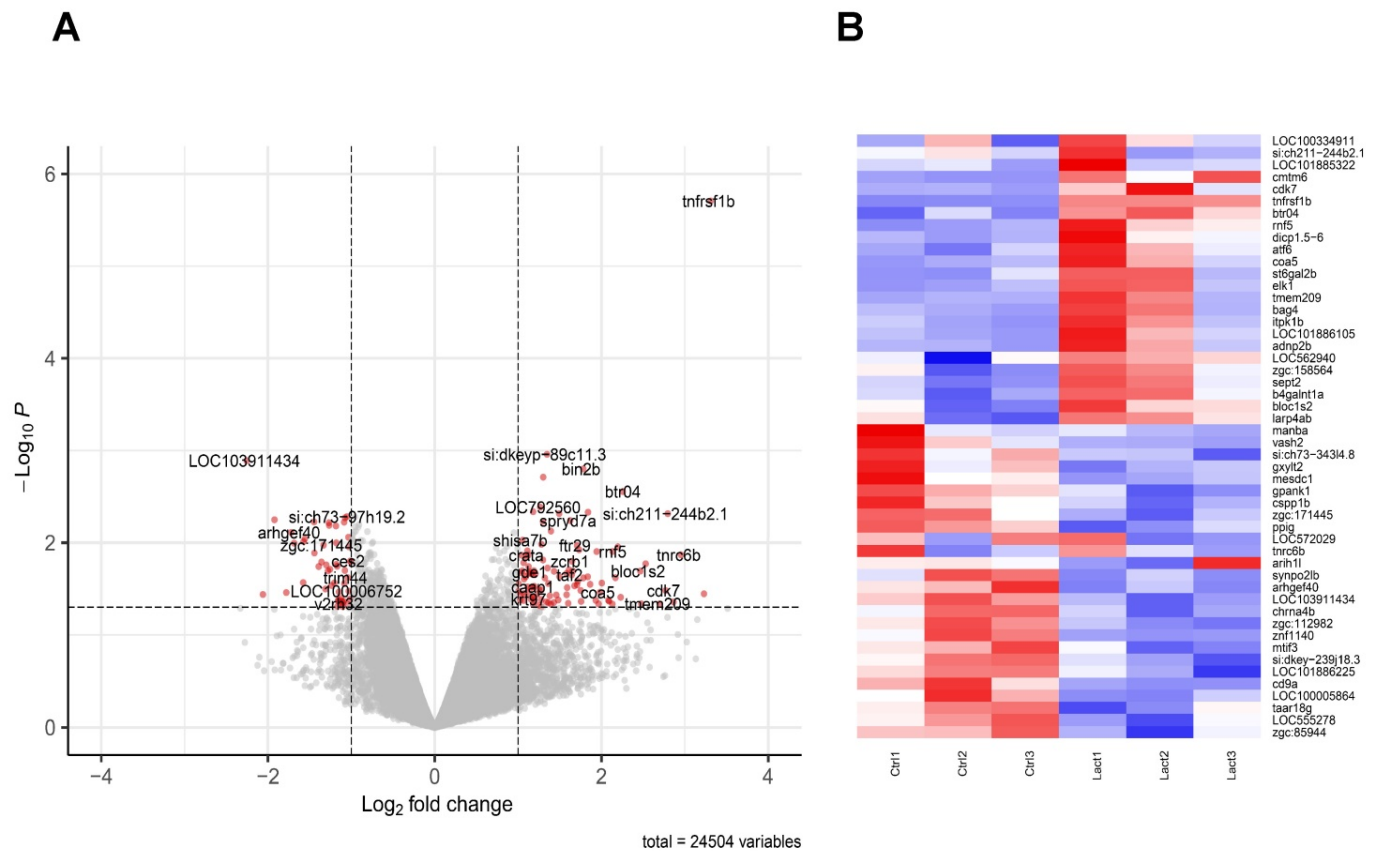
** Transcriptomic effect of lactate on macrophages in the regenerating caudal fin.** (A) Volcano Plot displaying the differentially expressed genes in macrophages upon lactate treatment (red dots). (B) Heatmap containing the top 25 significantly up-regulated and downregulated genes sorted by their average expression foldchange in macrophages from amputated caudal fins with (Lact) or without (Ctrl) lactate treatment; 3 biological replicates for each condition.

To draw a cartography of lactate-induced modifications, we isolated macrophages submitted to amputation, in presence or absence of lactate and performed and global analysis of mRNA. As the peak of TNFα-producing macrophages site was observed at 6 hpA at the amputation, we focused our microarray analysis at 24 hpA to identify downstream signaling pathways regulated by TNFα. To do so, we sorted macrophages by FACS from regenerating caudal fins at 24 hpA in a solution with or without 100 μM of L-lactate. Their corresponding mRNA were hybridized to Affymetrix 1.0 st microarrays to generate the expression profile for each condition. We then performed differential expression analysis to generate the transcriptomic signature of the lactate effect on macrophages. We found a list of 454 differentially expressed genes (P-value < 0.05) among them 190 were up-regulated and 264 down-regulated. Moreover, 104 up-regulated and 48 down-regulated genes displayed a foldchange greater than 2 (Figure 5A-B; Supplementary Table 1). We then performed gene ontology enrichment analysis on the DAVID platform to look for enriched pathways from the differentially expressed genes (Supplementary Table 2). We found a weak enrichment (P-value < 0.1) of pathways such as COPI-dependent Golgi-to-ER retrograde traffic (kifc1, sec22bb, kif5ab), Ephrin signaling (myl12.2, efnb2a, nck2a) and Activation of Matrix Metalloproteinases (cela1.1, prss1, si:dkey-239J18, 3).

Importantly, Tumor Necrosis Factor Receptor Superfamily, Member 1B (tnfrsf1b) outscored all up-regulated genes in terms of p-value and most genes in terms of foldchange. Tnfrsf1b, the ortholog of TNFR2 by opposition to TNFR1 is almost exclusively expressed in immune cells, stimulates NFκB signaling and has no intrinsic cell death inducing activity. We hypothesize that tnfrsf1b might play as a positive feedback loop to enhance survival and a pro-inflammatory phenotype 46.

Overall, the modification of macrophage transcriptomic profile induces key modifications in macrophage properties, enhancing phagocytosis, matrix degradation and cell motility while limiting apoptotic pathways.

## Discussion

In this study, we provide the first evidence that lactate influences macrophage behavior, recruitment and polarization during regenerative processes. An increase of lactate pathway follows the tissue amputation to coordinate metabolic changes in macrophage and inflammatory response. The modulation of gene expression adapts macrophage properties to face the challenge of blastema formation.

The role of lactate on inflammation has long been a puzzling question. While our study highlighted its activator function on macrophage recruitment and polarization, many studies pointed out the anti-inflammatory role of lactate, particularly in the context of cancer 47 48. The apparent discrepancy relies on the complexity of inflammation time-course during the regeneration process. Exogenous lactate supplementation increased the frequency of pro-inflammatory macrophages expressing *TNFa* only when administrated during the 24 hours following amputation. This suggests that lactate uptake is determinant during the proliferative phase of the blastema, when cells rely on very high energy requirements. Lactate may have a different impact on zebrafish macrophages throughout the last 48 hours of regeneration process. During this phase of differentiation, lactate could conversely promote a switch to a non-inflammatory macrophage phenotype 49.

While the present study illustrates the importance of lactate to coordinate inflammation and regeneration, it raises a new question regarding the origin of this metabolite upon amputation. The global administration of lactate and chemical inhibitors illustrated the role of lactate pathway on macrophage reprogramming but did not distinguish the impact of intra- or extra- cellular glycolysis and lactate production. scRNA-seq evidenced that macrophages are submitted to intracellular adaptions to modulate glycolysis and lactate metabolism. These modifications called for a cell-autonomous metabolic reprogramming. However, we demonstrated that a disruption of lactate uptake mediated by MCT1 depletion prevented macrophage answer at 24 hpA, suggesting the importance of an external supply of lactate. Here again, the discrepancy could be explained by the existence of successive phases during regenerative process. The sources of lactate that initiate and maintain macrophage metabolic reprogramming may change over time. This question calls for further investigations to consider other blastema cells and their metabolic dialogue with macrophage.

The activation of macrophages by lactate implies a broad modification of gene expression. This transcriptional transformation may be driven by a direct action of lactate on chromatin acetylation. Indeed, the subtle balance of histone acetylation was recently identified as an important mediator of lactate effect in regenerating context 45. As the coactivator of Histone acetyltransferase p300, HIF1a may play a determinant function in macrophage fate. Indeed, HIF1a is known to be activated by lactate 43 and to modulate macrophage motility during wound healing or to induce TNFa expression 51. The combination of HIF1a and histone acetyl transferase regulation may represent important effectors of lactate to modify transcriptomic profile in macrophages and coordinate metabolic changes and inflammatory response during regeneration.

The cartography of transcriptional modifications highlighted the main targets of lactate-induced reprogramming. Among them, Tnfrsf1b appears as the main target of lactate modulation. Tnfrs1b, one of the main receptors for Tnfa signaling, lack death-domains (TRADD and FADD) which allows to avoid direct activation of cell death and in turn induces the expression of inhibitory apoptosis protein 1 and 2 (cIAPs), the NF-κB pathway and PI3k-akt pathway promoting cell survival. Its overexpression might translate into a pro-inflammatory phenotype maintenance while avoiding apoptosis 53. Consistently, RNAseq profiling of human M1/M2 macrophages 54 display Tnfrs1fb ortholog (TNFR2) among the M1 markers in terms of p-value and fold-change, this expression was further validated in zebrafish larvae as a part of macrophage population core transcriptomic network 55. The exploration of lactate effectors also found Tumor necrosis factor receptor superfamily member 6 (fas), another TNF receptor, which although classically linked to apoptosis it has been described as an inducer of pro-inflammatory mediators and cytokines through ERK/JNK-dependent activation of Nfk-B in mammalian macrophages in vitro 56, 57. Consistently, we found an up-regulation of the colony-stimulating factor 3 a (csf3a) a chemokine involved in M1 macrophages polarization 58. On the other hand, we found Tumor Necrosis Factor Receptor Superfamily, Member 11 (tnfrs11b), a tnf receptor involved in the apoptosis of osteoclasts, and C-X-X motif chemokine Receptor 4 (Cxcr4) an important activator of an anti-inflammatory M2 like macrophages among the downregulated genes, suggesting once again maintenance of the pro-inflammatory program as well as macrophage survival promotion. Among the up-regulated genes we find zgc:174153 (cathepsin L2 family member) and cathepsin l (ctsl), genes actively involved in protein degradation in lysosomes. Moreover, bridging integrator 2b (bin2b), a phagosome formation mediator, as well as C3a anaphylatoxin chemotactic receptor 1 (c3ar1), a key component of complement cascade which induces neighboring cells apoptosis, displayed an up-regulation under lactate treatment, suggesting an improvement in macrophages phagocytic capacity. Interestingly, motor proteins such as kinesin-like protein c1 (kifc1), kinesin-like protein 5ba (kif5ba) and kinesin family member 20Bb (kif20bb), which are widely involved in cell protrusion formation 59, 60, were up-regulated while adhesion cellular genes such as Integrin beta 4 (itgb4), endothelial cell-adhesion molecule a (esama), integrin beta like 1 (itgbl1), delta-like protein (jag2b) and protocadherin 2 alpha b 10 (pcdh2ab10) decreased in presence of lactate, this might therefore be translated into an increase on macrophages motility.

Overall, this transcriptomic profile suggests that lactate treatment improve macrophages functions such as intracellular trafficking, phagocytosis, matrix degradation and cell motility while keeping an inflammatory phenotype and avoiding apoptosis. These modifications could lead to a better macrophage performance in order to help different processes such as tissue clearance and remodeling during zebrafish caudal fin regeneration.

## Supplementary Material

Supplementary figures.

Supplementary table 1.

Supplementary table 2.
