# Supplementary material for: Lactate metabolism coordinates macrophage response and regeneration in zebrafish
Source: Theranostics. 2022 May 13;12(8):3995–4009. doi: 10.7150/thno.65235 (PMC9131269; doi:10.7150/thno.65235)

Supplementary figures

Figure S1. Identification of key pathways modulated within macrophages of the regenerating caudal fin from Single-cell RNAseq data. (A) Dimensional reduction plots of the different cells populations from injured (cut) and uninjured (uncut) conditions (B) Macrophage markers-based sub-clustering from the myeloid cluster. (C) Metabolic gene network from the IPA analysis. (D) Violin plots of the number of features detected in every cell, and the percentage of mitochondrial genes after quality control assessment.

Figure S2. Glycolytic and lactate inhibitors alter the recruitment and the macrophage barrier. (A) Glycolysis and lactate metabolism inhibitory drugs. (B) Z projection of confocal images illustrating macrophage recruitment and migratory behavior at 6 hpA after amputation and immersion with zebrafish water (CT), 2DG (50 µM), DCA (500 µM), or GAL (500 nM) (Scale bar = 60 µm). (C) Z projection of confocal images illustrating macrophage recruitment and migratory behavior at 24 hpA after amputation and immersion with zebrafish water (CT), 2DG (50 µM), DCA (500 µM), or GAL (500 nM) (Scale bar = 60 µm). (D) Experimental design of macrophage barrier quantification and corresponding graph showing the quantification of the number of *mpeg*$^{+}$ macrophages at the fin tip at 6 hpA after amputation and immersion with zebrafish water (CT), 2DG (50 µM), DCA (500 µM), or GAL (500 nM) (mean ± SEM, n > 30, ordinary one-way ANOVA, Dunnett's multiple comparisons test, compared to control, ***p ≤ 0.001).

Figure S3. Glycolytic and lactate inhibitors impact on the caudal fin regeneration after amputation. (A) Diagram of the zebrafish larva caudal fin regeneration after amputation at 3 dpf. Wound epithelium forms at the fin tip after amputation and persists until 6 hpA.

Regenerative hyperproliferative blastema take place at 24 hpA. Then, gives way to the mesenchyme cells elongation, the new member morphogenesis, at 48 hpA. Finally, the new member equivalent to the original appears at 72 hpA. (B) Representative images of caudal fin regeneration at 72 hpA (Scale bar = 100 µm) with the corresponding graph showing the fin length or area at 72 hpA, after amputation and caudal vein injection with PBS (CT), 2DG (50 µM), DCA (500 µM), or GAL (500 nM) (mean ± SEM, n > 30*,* ordinary one-way ANOVA, Dunnett's multiple comparisons test, compared to control, ****p ≤ 0.0001). (C) Graph showing the fin length at 72 hpA, after amputation and immersion with zebrafish water (CT), immersion with OXA (10 mM) or caudal vein injection with OXA (10 mM) (mean ± SEM, *n < 30,* Kruskal-Wallis, Dunn's multiple comparisons test, compared to control, **p ≤ 0.01, ****p ≤ 0.0001). (D) Blastema cell proliferation after amputation and immersion in zebrafish water (CT), 2DG (50 µM), DCA (500 µM), GAL (500 nM) or OXA (10 mM). Anti-H3P antibody staining of the cells in the fin at 24 hpA, expressed as fold change cut/uncut (mean ± SEM, *n < 30,* Kruskal-Wallis, Dunn's multiple comparisons test, compared to control, *p ≤ 0.05, **p ≤ 0.01, ***p ≤ 0.001).

Figure S4. MCT1 genetic knockdown impacts macrophage polarization (A) Schematic representation of MCT1 (NM_20085) and MCT4 (NM_212708) main isoforms in zebrafish with mapping of guideRNA on the different exons. (B) Summary of gRNA sequences and features**.** (C) Graph showing the quantification of the number of $mpeg^{+}tnfa^{+}$ macrophages in the entire fin at 24 hpA after amputation of uninjected larvae, of scrambled larvae, of Mct1 -/- larvae, or Mct4 -/- larvae (mean ± SEM, *n < 30,* Kruskal-Wallis, Dunn's multiple comparisons test, compared to control, *p ≤ 0.05). (D) Graph showing the relative number of $mpeg^{+}tnfa^{+}$ macrophages (fold change of $mpeg^{+}tnfa^{+}$ macrophages over the total number of $mpeg^{+}$ macrophages macrophages) in the entire fin at 24 hpA after amputation of uninjected

larvae, of scrambled larvae, of Mct1 -/- larvae, or Mct4 -/- larvae (mean ± SEM, $n < 30$, Kruskal-Wallis, Dunn's multiple comparisons test, compared to control, non-significant).

Figure S5. Exogenous lactate impacts macrophage polarization and accelerates blastema cells proliferation. (A) Graph showing the quantification of the number of *mpeg*$^{+}$*tnfa*$^{+}$ macrophages in the entire fin at 6 hpA after amputation and immersion with zebrafish water (CT) lactate (100 µM) or GAL (500 nM) and lactate (100 µM) added at the same time (mean ± SEM, $n < 30$, Mann Whitney test, two-tailed, non-significant). (B) Graph showing the quantification of the number of *mpeg*$^{+}$*tnfa*$^{+}$ macrophages in the entire fin at 24 hpA after amputation and immersion with zebrafish water (CT) lactate (100 µM) or GAL (500 nM) and lactate (100 µM) added at the same time (mean ± SEM, $n < 30$, Mann Whitney test, two-tailed, non-significant). (C) Graph showing the relative number of *mpeg*$^{+}$*tnfa*$^{+}$ macrophages (fold change of *mpeg*$^{+}$*tnfa*$^{+}$ macrophages over the total number of *mpeg*$^{+}$ macrophages macrophages) in the entire fin at 6 hpA after amputation and immersion with zebrafish water (CT), lactate (100 µM) or GAL (500 nM) and lactate (100 µM) added at the same time (mean ± SEM, $n < 30$, Mann Whitney test, two-tailed, non-significant). (D) Graph showing the relative number of *mpeg*$^{+}$*tnfa*$^{+}$ macrophages (fold change of *mpeg*$^{+}$*tnfa*$^{+}$ macrophages over the total number of *mpeg*$^{+}$ macrophages macrophages) in the entire fin at 24 hpA after amputation and immersion with zebrafish water (CT), lactate (100 µM) or GAL (500 nM) and lactate (100 µM) added at the same time (mean ± SEM, $n < 30$, Mann Whitney test, two-tailed, non-significant (E) Blastema cell proliferation after amputation and immersion in zebrafish water (CT), lactate (100 µM) or GAL (500 nM) and lactate (100 µM) added at the same time. Anti-H3P antibody staining of the cells in the fin at 6 hpA, expressed as fold change cut/uncut (mean ± SEM, $n < 30$, Mann Whitney test, two-tailed, $*p \leq 0.05$).

Supplementary figure 1

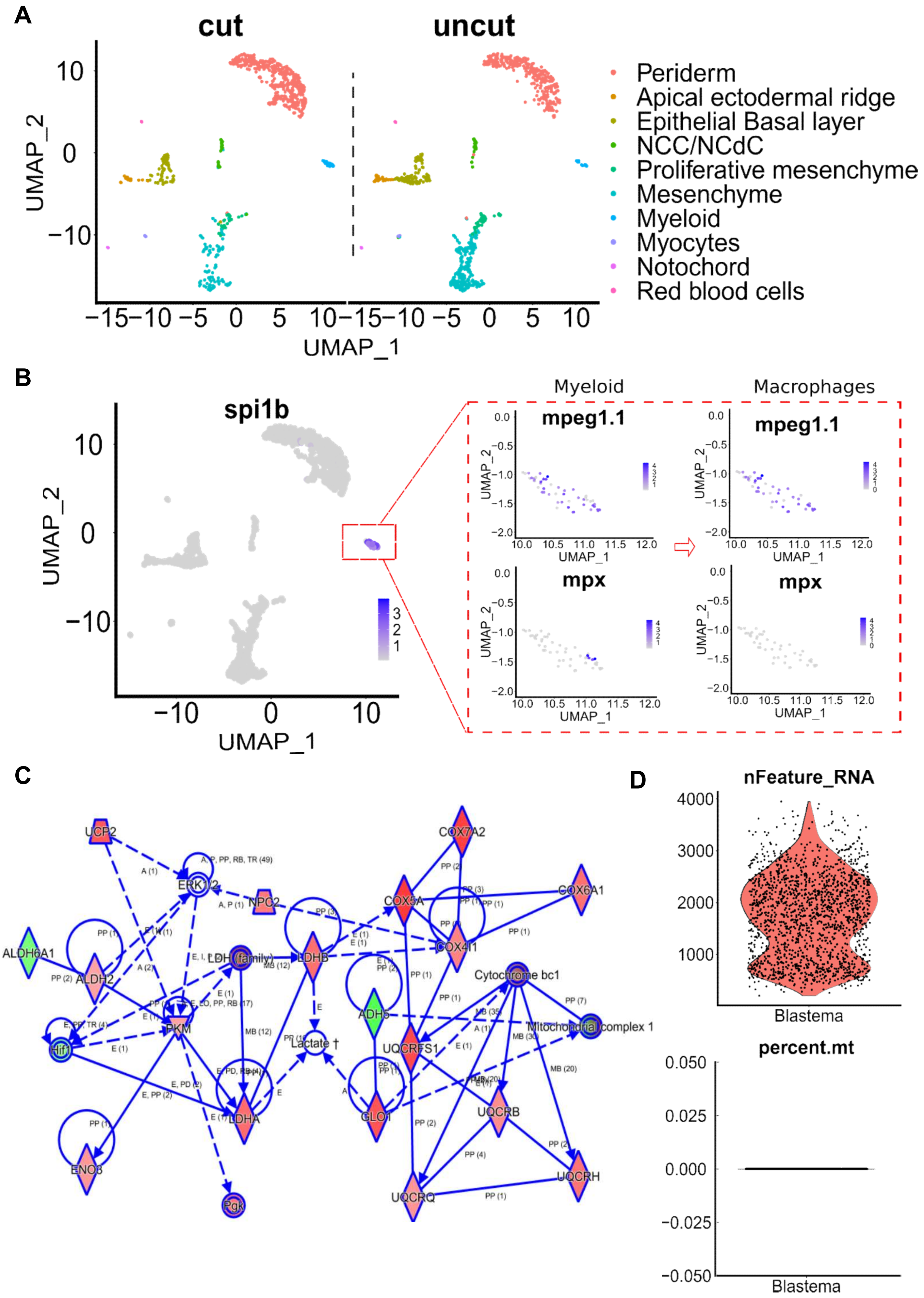

Supplementary figure 2

A

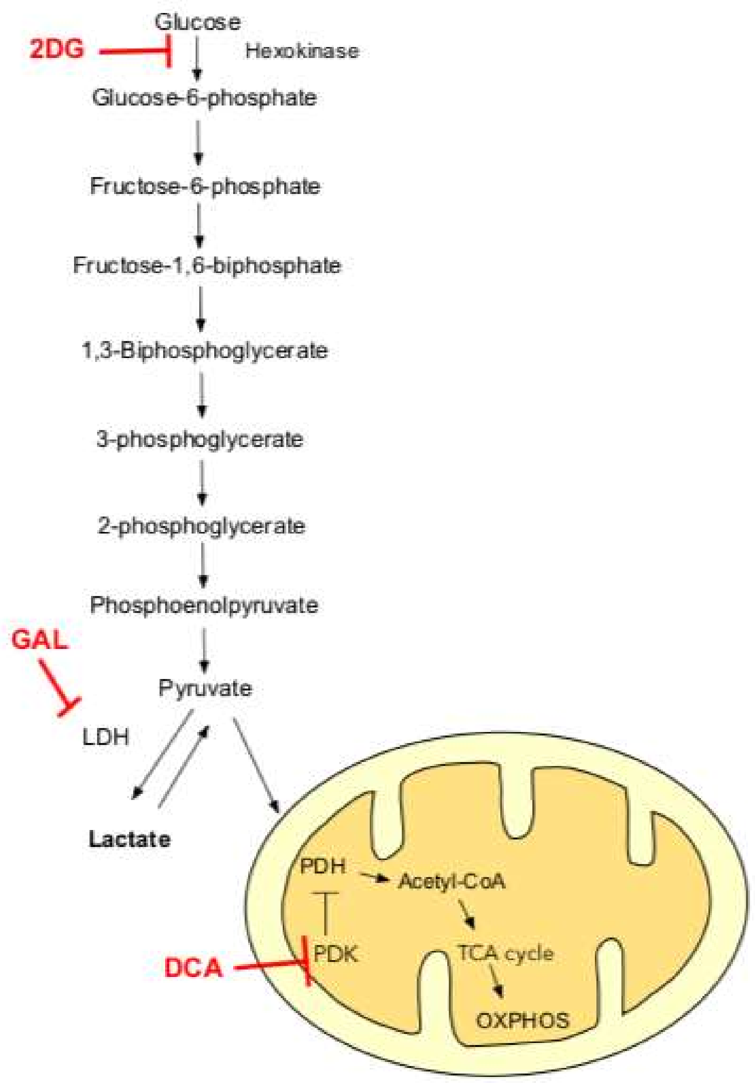

B

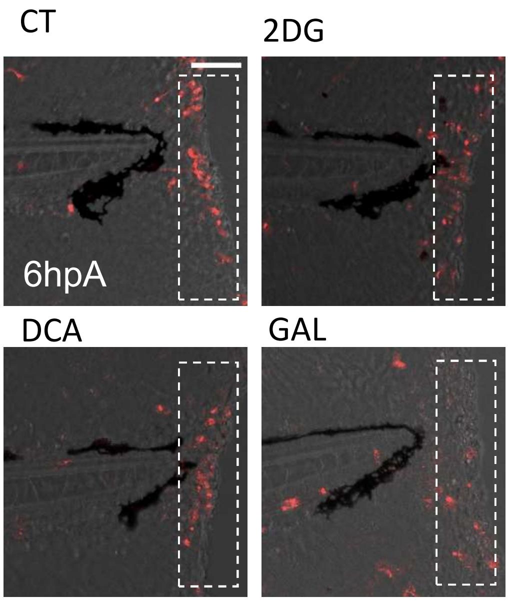

C

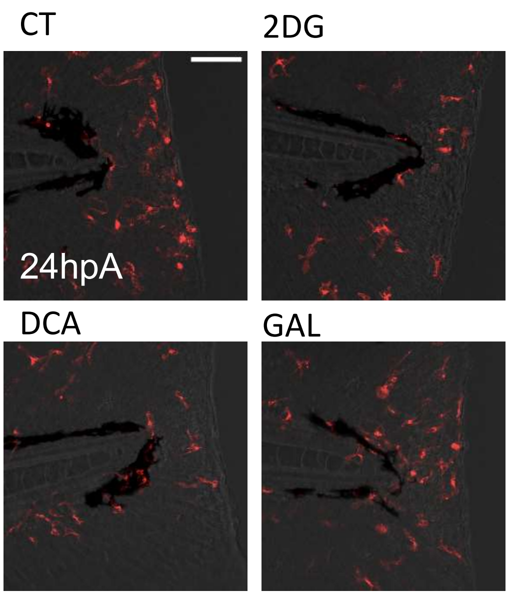

D

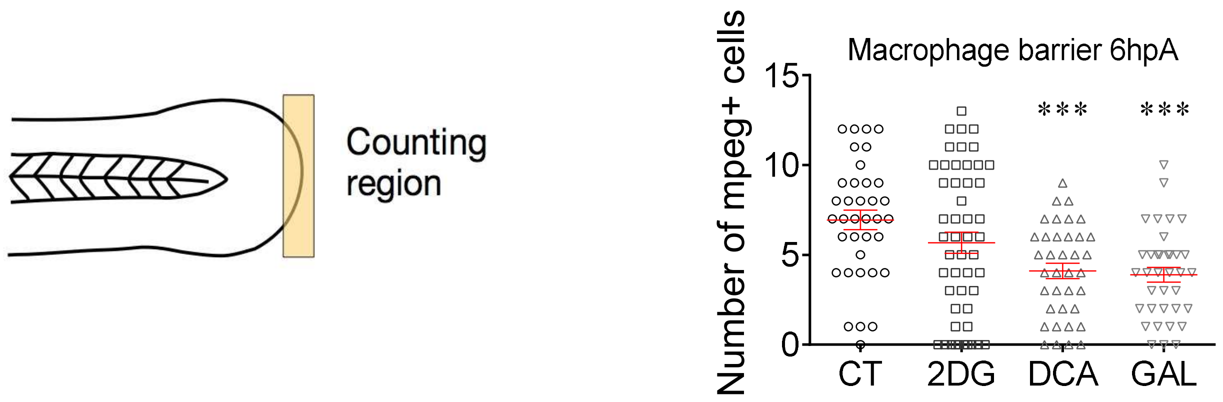

Supplementary figure 3

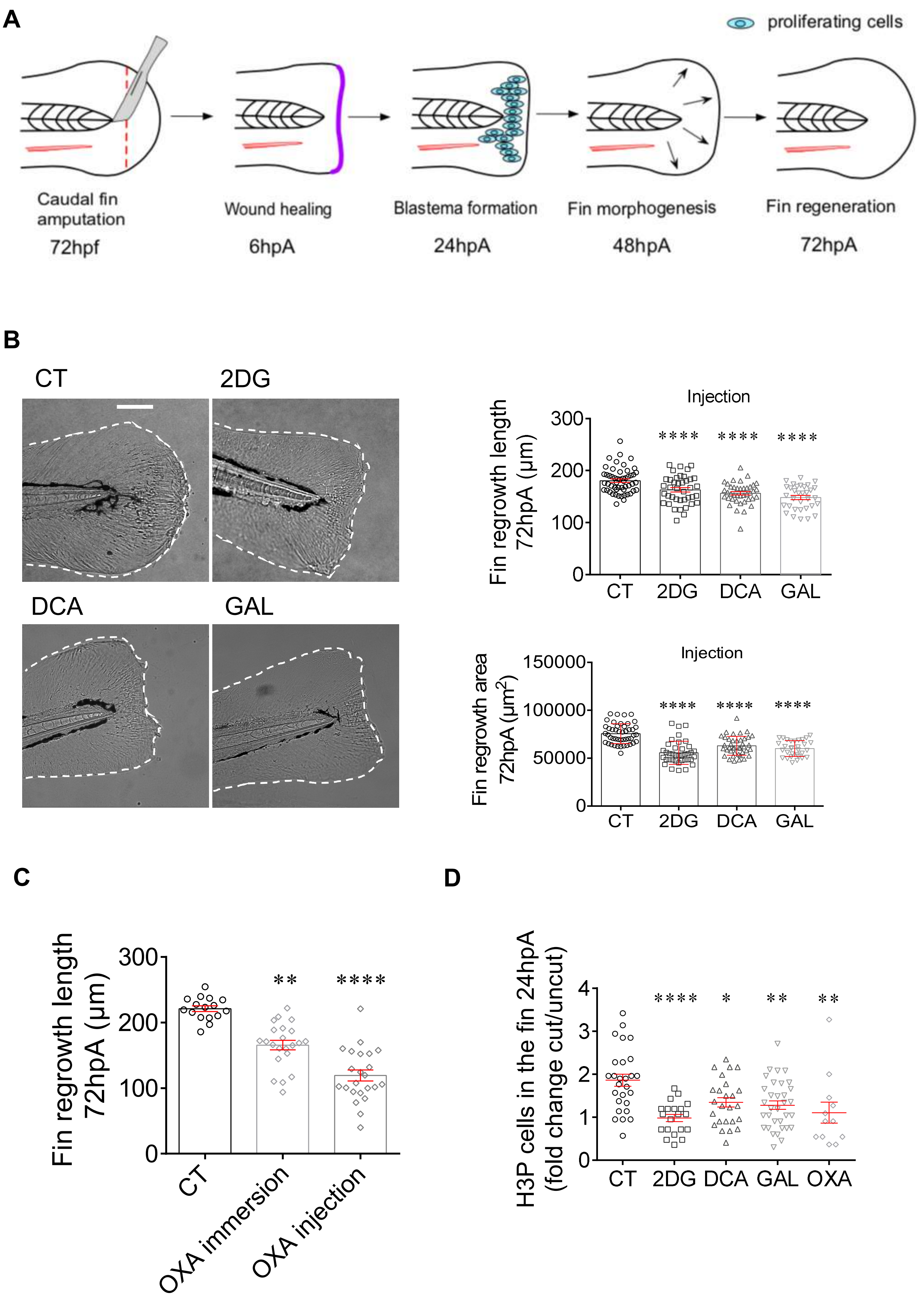

Supplementary figure 4

A

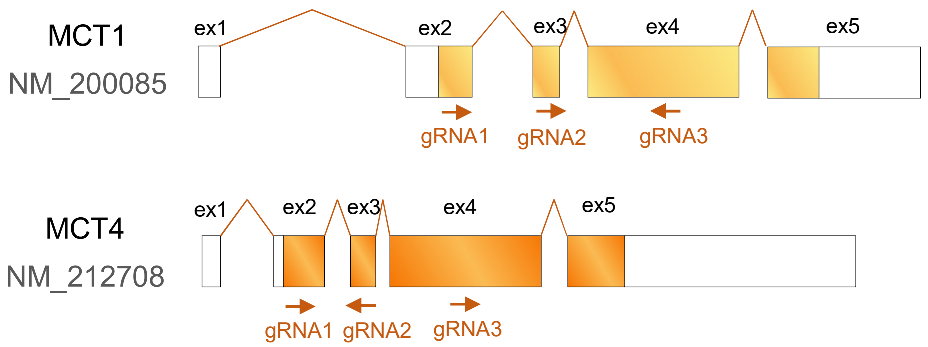

B

Gene	RefSeq	N°	Strand	Targeted Exon	Target Sequence + PAM	gRNA sequence
		gRNA1	+	2	**GGTGTTCTTCAAGGAGATTGAGG**	GGTGTTCTTCAAGGAGATTG
**MCT1**	NM_200085	gRNA2	+	3	**CGGCCAATCATGATCGCTGGAGG**	CGGCCAATCATGATCGCTGG
		gRNA3	-	4	**TTTGCCACCAGACCCATAGACGG**	TTTGCCACCAGACCCATAGA
		gRNA1	+	2	**CTTATCCGGGAGTTTGGAGTGGG**	CTTATCCGGGAGTTTGGAGT
**MCT4**	NM_212708	gRNA2	-	3	**GACACCCAAATCGGTTCACTAGG**	GACACCCAAATCGGTTCACT
		gRNA3	+	4	**TTCACCGTCTTCAAAGATCGTGG**	TTCACCGTCTTCAAAGATCG
		gRNA1				CGTTAATCGCGTATAATACG
**Scrambled**	N.A.	gRNA2			Random sequence designed by Kroll et al, elife - 2021	CATATTGCGCGTATAGTCGC
		gRNA3				GGCGCGTATAGTCGCGCGTA

C

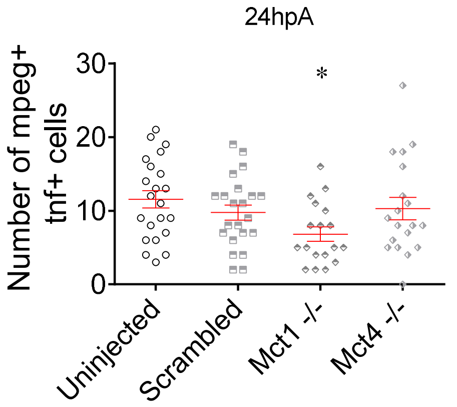

D

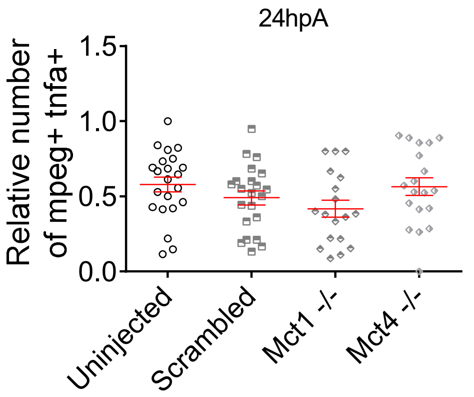

Supplementary figure 5

A

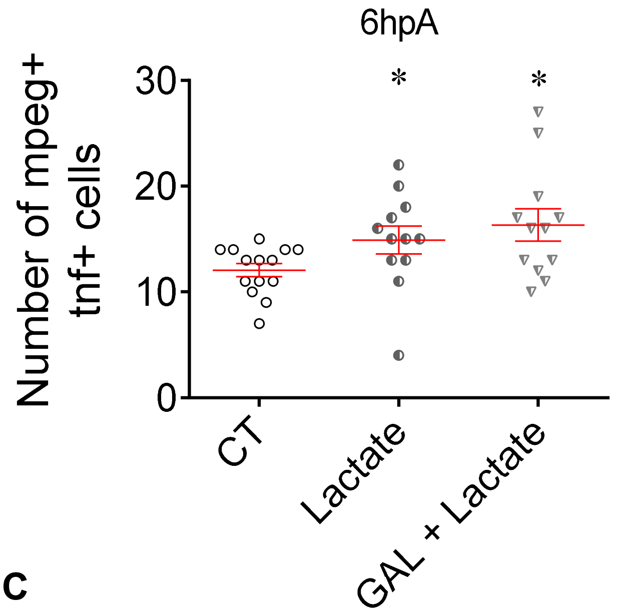

B

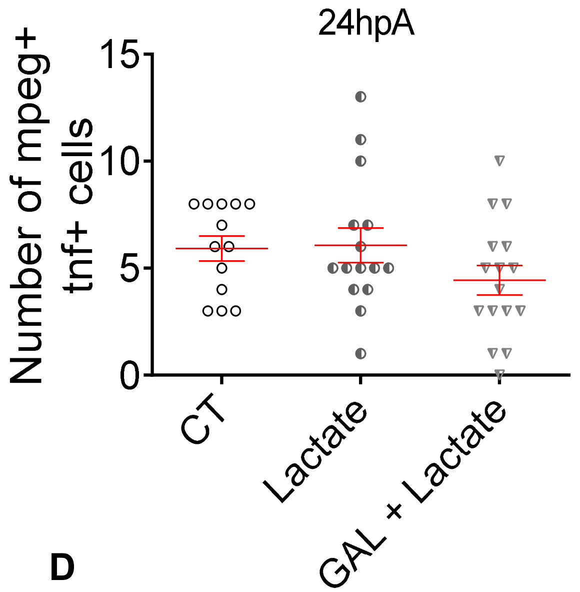

C

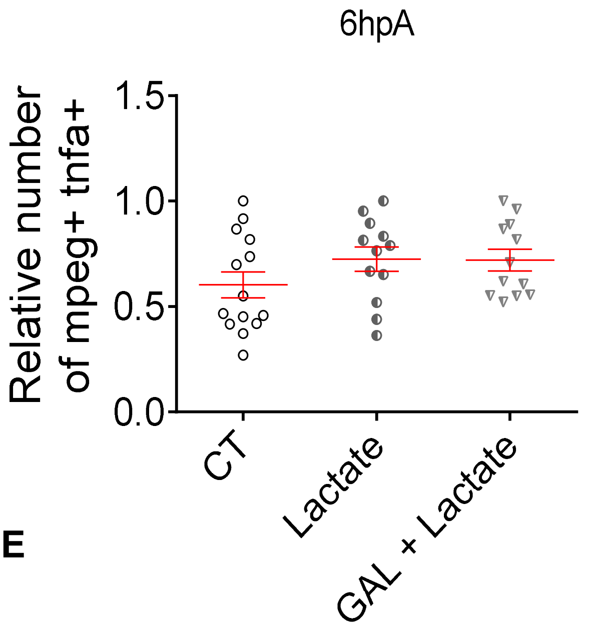

D

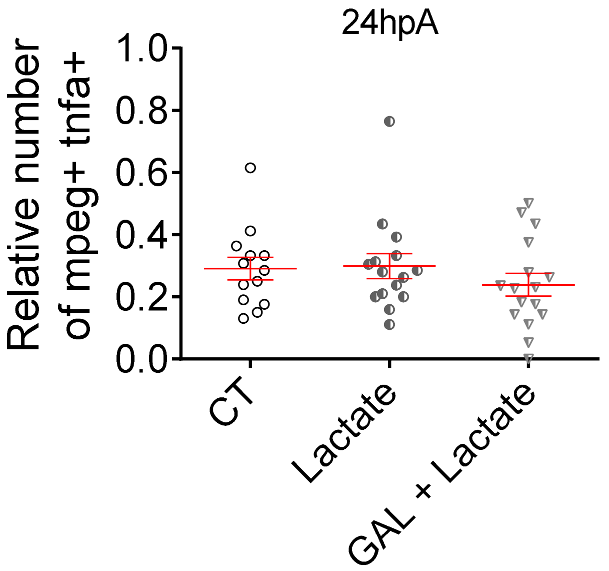

E

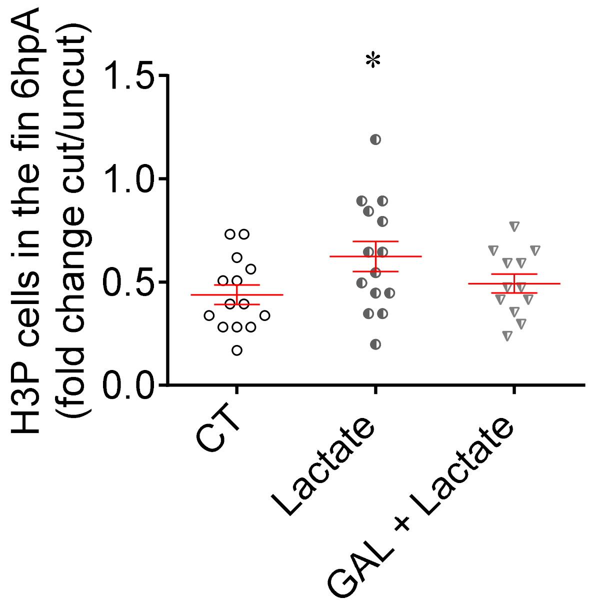

Supplement: Supplementary file 1 — Supplementary figures. [file thnov12p3995s1.pdf]
